# Efficacy of Three Light Technologies for Reducing Microbial Populations in Liquid Suspensions 

**DOI:** 10.1155/2014/673939

**Published:** 2014-03-04

**Authors:** Angeliki Birmpa, Apostolos Vantarakis, Spyros Paparrodopoulos, Paul Whyte, James Lyng

**Affiliations:** ^1^Environmental Microbiology Unit, Department of Public Health, Medical School, University of Patras, Rio, 26500 Patras, Greece; ^2^School of Veterinary Medicine, University College Dublin, Belfield, Dublin 4, Ireland; ^3^School of Agriculture and Food Science, University College Dublin, Belfield, Dublin 4, Ireland

## Abstract

The aim of the current study was to evaluate the effectiveness of three nonthermal light technologies (NUV-Vis, continuous UV, and HILP) on their ability to inactivate *Escherichia coli* K12 and *Listeria innocua.* *E. coli* K12 was selected as a representative microorganism for the enterohaemorrhagic foodborne pathogen *E. coli* O157:H7 and *L. innocua* as a surrogate microorganism for the common foodborne pathogen *Listeria monocytogenes*, respectively. The liquid matrix used for the disinfection experiments was a liquid matrix (MRD solution). The results of the present study show that the HILP treatment inactivated both *E. coli* and *L. innocua* more rapidly and effectively than either continuous UV-C or NUV-vis treatment. With HILP at 2.5 cm from the lamp, *E. coli* and *L. innocua* populations were reduced by 3.07 and 3.77 log_10_ CFU/mL, respectively, after a 5 sec treatment time, and were shown to be below the limit of detection (<0.22 log_10_ CFU/mL) following 30 sec exposure to HILP (106.2 J/cm^2^). These studies demonstrate the bactericidal efficacy of alternative nonthermal light technologies and their potential as decontamination strategies in the food industry.

## 1. Introduction

In recent years, nonthermal technologies have shown potential as alternatives to conventional pasteurization, with scope for inactivating pathogens and spoilage microorganisms without any of the adverse effects on product quality associated with thermal treatments such as reduced nutritional value or altered sensory attributes [1, 2].

Some pathogenic bacteria such as *Listeria monocytogenes* and other psychrotrophic bacteria can grow at low temperatures, threatening public health and shortening the shelf life of raw foods [3]. Many outbreaks associated with fresh ready-to-eat produce have been reported previously with *E. coli, Listeria, and Salmonella* identified as implicated pathogens [3–8].

Currently fresh produce, fruit, and vegetables, are washed with aqueous sanitizers such as chlorine, hydrogen peroxide, and trisodium phosphate in order to reduce the microbial load of fresh produce. However, the use of aqueous sanitizers alone has not been successful in controlling foodborne pathogens [9] and treatment of produce with chlorine has adverse effects, such as formation of trihalomethanes [10, 11]. Organic acids, mainly citric, lactic, and acetic acid, which are in GRAS (Generally Recognized As Safe) status, have been also used as disinfectants because of their bactericidal activity [12]. Hydrogen peroxide (H_2_O_2_), also referred to as hydrogen dioxide, has also been used as bleaching agent due to its strong oxidizing power [12]. As a result there is a need for the development of additional effective hurdles for these raw foods which can eliminate or significantly reduce microbial contamination, be environmentally friendly while not impacting on the quality of foods [4]. A range of nonthermal technologies (Ultrasound, UV-C, Ozone, and HHP) have already been successfully implemented on a number of ready-to-eat fruits and vegetables [11, 13–17].

NUV-vis light 395 ± 5 nm is a safe, non-UV based decontamination technology which is thought to act by stimulating endogenous microbial porphyrin molecules to produce oxidizing reactive oxygen species (ROS), predominantly singlet oxygen (^1^O_2_) that damages cells leading to microbial death [18–21]. Exposure of microorganisms to visible light particularly at wavelengths of 405 nm, has been shown to be effective in inactivating a range of bacteria, including Gram- positive and Gram-negative bacterial species and antibiotic-resistant microorganisms such as Methicillin-resistant *Staphylococcus aureus*, and its use has been suggested for a range of decontamination applications [22–26].

The inactivation mechanism of UV light is the formation of photoproducts in the DNA of target microorganisms. Of these photoproducts, the most important is the pyrimidine dimer which forms between adjacent pyrimidine molecules on the same strand of DNA and can interrupt both DNA transcription and translation [27]. The DNA damage inflicted by UV-C radiation leads to cell death by altering the microbial DNA through dimer formation between neighbouring pyrimidine nucleoside bases in the same DNA strand [28, 29].

High-intensity light pulses (HILP) is a nonthermal technology which uses short (100–400 *μ*s) high-power pulses of broad-spectrum (200–1100 nm) and has been used to inactivate bacteria (vegetative cells and spores), yeasts, moulds, and even viruses [30, 31]. The mode of action of HILP on microorganisms is likely the photochemical action of the UV-C part of the light spectrum that causes thymine dimerization in the DNA chain preventing replication and ultimately leading to cell death [2, 32–34]. Microbial inactivation using HILP has gained attention in recent years due to lower energy consumption compared to conventional thermal processes [35]. Depending on the energy delivered through each flash, the distance between the lamps and the contaminated matrix, the targeted microorganism, and even the nature of the contaminated matrix itself, HILP has been reported to result in a 0.5 to 8 log⁡_10_⁡ CFU/mL bacterial reduction [36]. The germicidal action of HILP has been also attributed to the localized elevated temperature due to the UVs and IR radiations leading to bacterial disruption [33, 37–40].

The objective of the present work was to evaluate the effectiveness of three nonthermal light technologies (NUV-vis, Continuous UV, and HILP) to reduce microbial populations in a liquid matrix. Different treatment intensities and times were selected in order to investigate the inactivation capacity of each light technology on one Gram-negative (*E. coli *K_12_) and one Gram- positive bacteria (*L. innocua *NCTC 11288). *E. coli* K12 and *L. innocua *were selected as surrogate organisms for *Escherichia coli* O157:H7 and *Listeria monocytogenes*, respectively [2]. To the authors' knowledge, this is the first paper where all these three nonthermal light technologies were compared for their ability to inactivate possible foodborne pathogens.

## 2. Material and Methods

### 2.1. Microorganisms and Culture Preparation

Experiments were conducted using *E. coli* K_12_ (DSM 1607) and *L. innocua *(NCTC 11288). The strains were maintained at 4°C on Tryptone Soya Agar, TSA (Oxoid, Hampshire, UK). For inoculation of the model solutions, cultures of *E. coli* or *L. innocua* grown overnight at 37°C in Tryptone Soya Broth, TSB (Oxoid), were used. The 24 h cultures were then centrifuged for 10 min at 30,000 ×g and the resulting pellets were washed and centrifuged twice in Maximum Recovery Diluent (MRD, Oxoid) before being mixed together by resuspending in a final volume of 10 mL MRD. This resulted in mixed culture cell suspensions of ~10^8^ colony forming units per milliliter (CFU/mL). The suspensions containing both *E. coli* and *L. innocua* inoculates were assessed for susceptibility to three light technologies in a liquid matrix (MRD). Mixed pure cultures (in MRD) were prepared as described previously. Samples (10 mL) were then placed into Petri dishes (50 mm diameter). They were then positioned at different distances from the lamp source. After removal of covers, Petri dishes containing the MRD solutions were subjected to different light doses ranging from 0.18 to 106.2 J/cm^2^.

### 2.2. UV Equipment

#### 2.2.1. NUV-Vis Light Unit

The NUV-vis light was produced by a light-emitting diode (LED) array (OD-2049) (Opto Diode Corp, sourced from AP Technologies, Bath, UK) with a central wavelength of 395 ± 5 nm, a bandwidth of 12 nm full-width at half maximum (FWHM), and a half intensity beam angle of 30° [40]. The irradiance (J∗cm^−2^) of light emitted from the LED unit was measured using a UV-VIS Radiometer (model no. RM12, Dr. Gröbel UV Electronik, GmbH, Ettlington, Germany) fitted with a RM UV-A sensor (part no. 811030, Dr. Gröbel UV Electronik). Distances of 3, 12, and 23 cm from the light source were chosen for treatments. The corresponding energy intensities and time needed to achieve them are presented in Table 1. These distances represented the most extreme to the least extreme treatments according to the study of Haughton et al. [41]. Construction of the LED unit was as previously described [41]. Sample temperatures were measured during the treatment using a K-type thermocouple attached to a Grant Data Logger (Squirrel 2040; Grant Instruments) to ensure that the maximum temperature reached was nonlethal to the bacteria under the treatment times investigated (<50°C) (Figure 6).

#### 2.2.2. Continuous UV Equipment

The UV unit was a custom-made unit with intimal dimensions (length × width × height) of 790 × 390 × 345 mm and consisting of four 95-W bulbs (Baro Applied Technology Limited, Athens, Greece) 500 mm in length. The UV dose (*D*) was calculated by using the following equation:

(1) $$\begin{array}{c} D:I_{254\;\text{nm}}*t, \end{array}$$

where *D* is the dose (J/cm^2^), *I*_254 nm_ is the dosage rate, and *t* is the retention time (in seconds). The UV dosages (J/cm^2^) were varied by altering the distance of the sample (6.5, 17, and 28.5 cm) from the light source and by changing the treatment time (Table 1). Sample temperatures were measured during the treatment using a K-type thermocouple attached to a Grant Data Logger (Squirrel 2040; Grant Instruments) to ensure that the maximum temperature reached was nonlethal to the bacteria under the treatment times investigated (<50°C) (Figure 7).

#### 2.2.3. HILP (High-Intensity Light Pulses) Unit

The HILP unit was a benchtop SteriPulse-XL system (Xenon, USA). The system comprised a high-energy pulsed ultraviolet-visible flash lamp (Type C, 190 nm spectral cut-off point) delivering a maximum of 1.27 J/cm^2^. The pulse width produced was 360 *μ*s at a fixed pulse rate of 3 Hz. The pulse energy delivered to the sample varied depending on its distance from the quartz window within the HILP chamber. The HILP dose of treatments applied in the present study was calculated in accordance with the manufacturer's instructions [42]. Distances of 2.5, 8, 11.5, and 14 cm were selected for treatments, in order to achieve a wide spectrum of dosages varying between 0.18 and 106.2 J/cm^2^. The corresponding dosages and time needed to achieve them are presented in Table 1. During HILP treatment, samples were placed in an iced bath to minimize heating. Sample temperatures were measured during the treatment using a K-type thermocouple attached to a Grant Data Logger (Squirrel 2040; Grant Instruments, Cambridge, UK) to ensure that the maximum temperature reached was nonlethal to the bacteria under the treatment times investigated (<50°C) (Figure 8).

### 2.3. Microbiological Analysis

After treatment of liquid samples, the contents of each Petri dish were transferred to sterile containers. Tenfold dilution series were prepared in MRD and 0.1 mL of each dilution was pour plated in duplicate using TBX (CM0945, Oxoid) for *E. coli* and *Listeria* Selective Agar (Oxford formulation, CM0856, Oxoid) for *L. innocua*. The plates were incubated at 44°C and 37°C for 24 and 48 h, respectively. Mean counts for each treatment were calculated and converted to log_10_ CFU/mL values with results for surviving numbers of microorganisms in MRD expressed per mL (CFU/mL). The plates were then used to enumerate viable cells in untreated controls and in samples following processing. The survival of bacterial cells following illumination was monitored by counting their viable number after exposure of the suspended bacteria to light. Bacterial cultures grown under the same conditions but without light exposure served as controls. The results were expressed as the logarithmic reduction (log⁡_10_⁡*N*/*N*_0_), where *N*_0_ is the initial microbial load and *N* the number remaining after treatment. All experiments were repeated at least three times.

### 2.4. Statistical Analysis

All experiments were carried out in triplicate. During each experiment two samples were taken at any time to conduct microbial counts. The microbiological data were analyzed in terms of log⁡_10_⁡(*N*/*N*_0_), where *N* is the microorganism load at a given time, and *N*_0_ corresponds to the initial microbial load of untreated samples. The data for inactivation of *E. coli* and *L. innocua* by NUV-vis light, continuous UV and HILP were analyzed for statistical significance using SPSS 21.0 (SPSS Inc., Chicago, USA). Results were compared by an analysis of variance followed by Tukey's pairwise comparison of the means with significance defined at the *P* < 0.05 level. Moreover, Pearson coefficient was used for measuring correlation between values.

## 3. Results

### 3.1. Inactivation Using NUV-Vis 395 ± 5 nm

**Figure 1:**
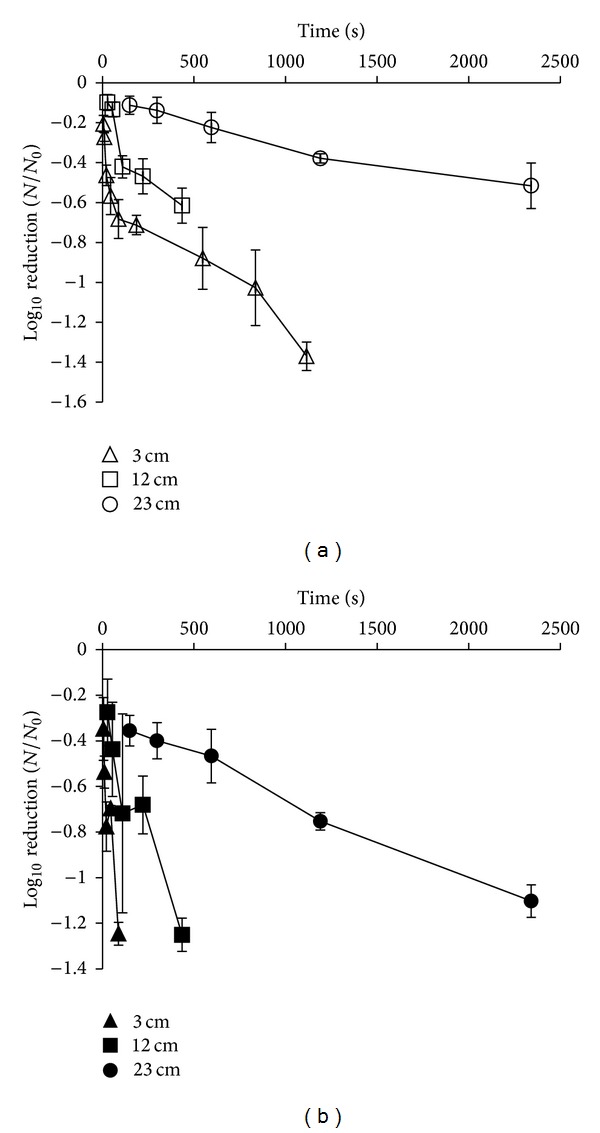
Survival curves of *E. coli* suspended in maximum recovery diluent (MRD) placed at 3 cm (∆), 12 cm (□), and 23 cm (○) and *L. innocua* placed at 3 cm (▲), 12 cm (■), and 23 cm (●) from the high-intensity near ultraviolet/visible (NUV-vis) 395 ± 5 nm light source (results expressed as mean log⁡_10_⁡ CFU/mL).

The inactivation rate of *E. coli* and *L. innocua *was dose dependent. Generally, it was observed that as the distance from the lamp was increased, the time needed for inactivation was longer (Figure 1). Corresponding doses delivered by this method are illustrated in Table 1.

When low dosages were implemented (0.18, 0.36, 0.72, and 1.44 J/cm^2^), the observed inactivation rates were similar for both *E. coli* and *L. innocua *(*P* > 0.05). However, when a higher dose of 2.832 J/cm^2^ was delivered, *L. innocua* exhibited a higher log reduction (1.25 log⁡_10_⁡ CFU/mL) compared to *E. coli* (0.68 log⁡_10_⁡ CFU/mL) after 88 sec of treatment (*P* < 0.05), around 2 times the inactivation log of the more resistant bacterium of *E. coli*. Moreover, when high dosages were achieved, the inactivation rates of *L. innocua* remained significantly higher with a maximum average log⁡_10_⁡ CFU/mL reduction of 2.74 achieved after 1115 sec of treatment, compared to that of *E. coli* where the maximum average log reduction after the same time was 1.37 log⁡_10_⁡ CFU/mL (*P* < 0.05). Moreover, the log reduction achieved for *L. innocua* remained higher than that of *E. coli* after the longest exposure time. At 23 cm from the light source, a higher susceptibility was observed for the *L. innocua* strain, giving a log reduction at the highest dose (2.832 J/cm^2^) of 1.10 log⁡_10_⁡ CFU/mL, significantly greater (*P* > 0.05) than the corresponding reduction for *E. coli *(0.52 log⁡_10_⁡ CFU/mL reduction). It has to be mentioned that temperatures remained below 50°C for all treatments used in the study.

### 3.2. Inactivation Using Continuous UV Light

**Figure 2:**
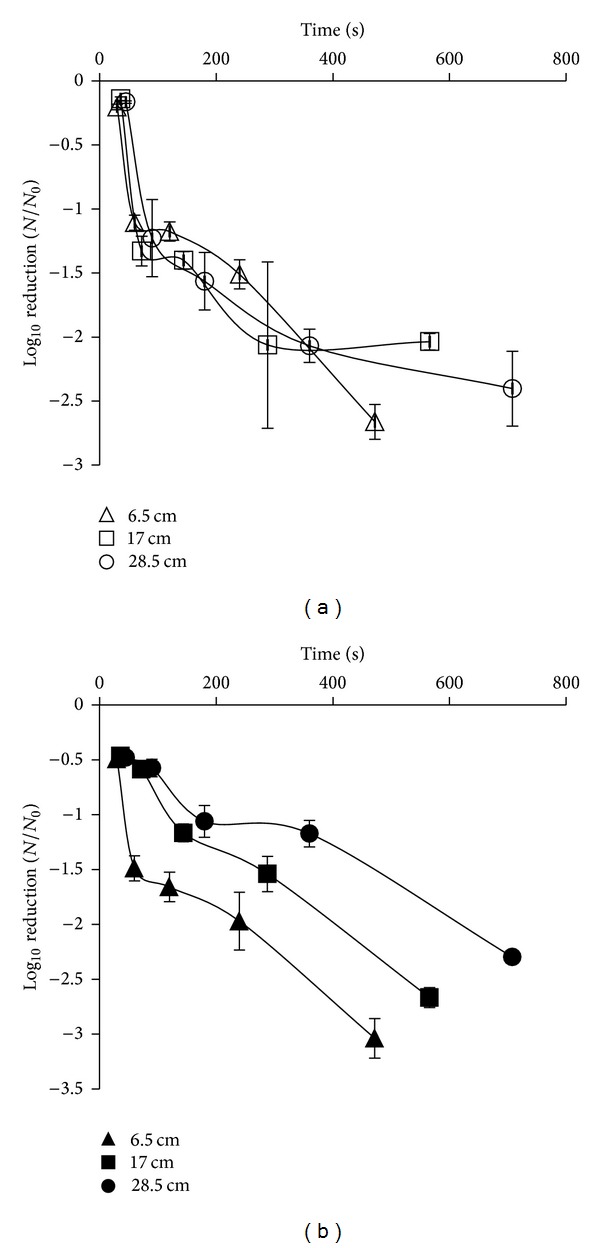
Survival curves of *E. coli* suspended in maximum recovery diluent (MRD) placed at 6.5 cm (∆), 17 cm (□), and 28.5 cm (○) and *L. innocua* placed at 6.5 cm (▲), 17 cm (■), and 28.5 cm (●) from continuous UV light source (results expressed as mean log⁡_10_⁡ CFU/mL).

There was an increase between dosage and log⁡_10_⁡ CFU/mL reduction of both microorganisms. Reductions of 2.66 log⁡_10_⁡ CFU/mL and 3.04 log⁡_10_⁡ CFU/mL were achieved for *E. coli *and *L. innocua,* respectively. The highest reductions were achieved at the shortest distance from the lamp (6.5 cm) and at the shortest treatment time (472 sec) (Figure 2) when a dose of 2.832 J/cm^2^ was implemented (Table 1). However, the susceptibility of two microorganisms when this light technology was used was not significantly different (*P* = 0.749). Temperatures remained below 50°C for all treatments used in the study.

### 3.3. Inactivation Using HILP

**Figure 3:**
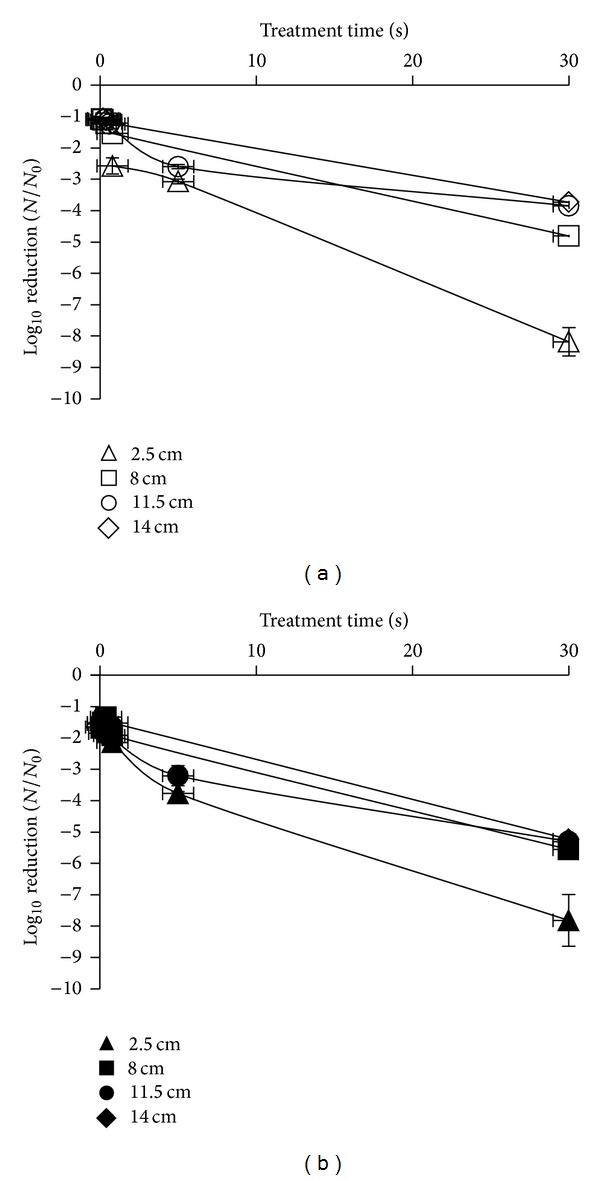
Survival curves of *E. coli* suspended in maximum recovery diluent (MRD) placed at 2.5 cm (∆), 8 cm (□), 11.5 cm (○), and 14 cm (◊) and *L. innocua* placed at 2.5 cm (▲), 8 cm (■), 11.5 cm (●), and 14 cm (♦) from HILP source (results expressed as mean log_10_ CFU/mL).

The measured dosages delivered with this light method and the time needed to achieve them is illustrated in Table 1. In general, increased dosage resulted in the greater reductions for both *E. coli* and *L. innocua*. The least susceptible microorganism was *E. coli* (Figure 3). A dosage of 17.7 J/cm^2^ resulted in log reductions of *E. coli* and *L. innocua *populations (3.07 and 3.77 log⁡_10_⁡ CFU/mL, resp.) and were both below the limit of detection (<0.22 log⁡_10_⁡ CFU/mL) following exposure to HILP at 106.2 J/cm^2^. When a dosage of 54 J/cm^2^ was implemented, reductions of 4.81 and 5.56 log⁡_10_⁡ CFU/mL were achieved for  *E. coli* and *L. innocua,* respectively. At a dosage of 36 J/cm^2^, a degree of variation was observed between the two tested microorganisms. For example, *E. coli* was reduced by 3.85 log⁡_10_⁡ CFU/mL, whereas *L. innocua* was reduced by 5.30 log⁡_10_⁡ CFU/mL (*P* < 0.05). The susceptibility of two microorganisms when this light technology was used was significantly different (*P* < 0.05). Temperatures did not exceed 50°C for any of the HILP treatments used in the current study.

### 3.4. Comparisons between Three Light Technologies

**Figure 4:**
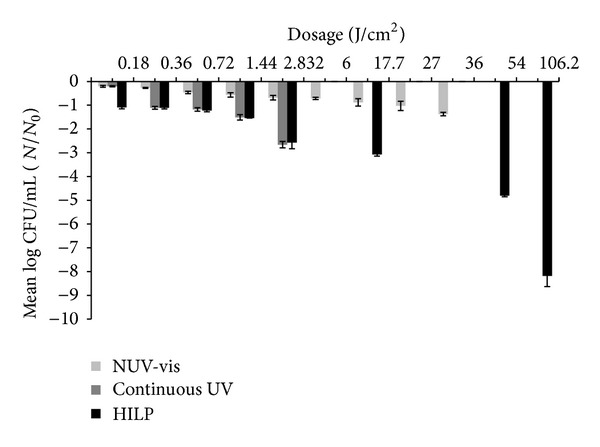
Mean log⁡_10_⁡ CFU/mL *E. coli* on MRD after treatment at the same dosages at shortest distance with 3 different light equipment.

**Figure 5:**
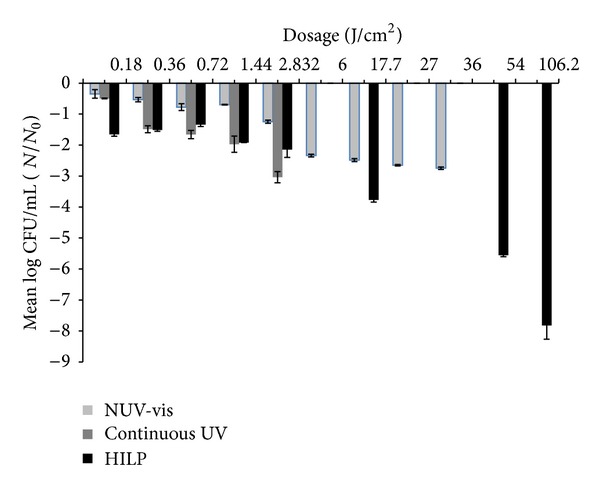
Mean log⁡_10_⁡ CFU/mL *L. innocua* on MRD after treatment at the same dosages at shortest distance with 3 different light equipment.

The three light technologies were tested for their disinfection capacity on two microorganisms (Figures 4 and 5). At low dosages (0.18, 0.36, and 0.72 J/cm^2^) the results between the two microorganisms, when the three light technologies were used, were all significant (*P* < 0.05). When 1.44 J/cm^2^ was implemented, the log⁡_10_⁡ (CFU/mL) reduction at NUV-vis light and continuous UV light for both microorganisms was significant (*P* < 0.05), whereas when comparisons with HILP light were done, the differences between the susceptibility of the tested microorganisms did not differ (*P* > 0.05). When 2.832 J/cm^2^ was implemented in both continuous UV light technology and HILP, the disinfection efficiency of *E. coli* and *L. innocua* did not differ significantly (*P* = 0.306 and *P* = 0.116, resp.). Finally, at higher dosages, the correlations between log_10_ (CFU/mL) reduction of NUV-vis and PUV, as the two organisms are concerned, were all significant (*P* < 0.05). Generally, it was observed that in all light technologies (NUV-vis, continuous UV, and HILP) a significant correlation (*P* < 0.05) between doses and microbes log reduction existed (Figures 4 and 5).

## 4. Discussion

The current study demonstrated that both *E. coli* and *L. innocua* are susceptible to all three light technologies investigated. Previous studies have investigated the lethal effects of high-intensity ultraviolet 405 nm light on *Escherichia*, *Salmonella*, *Shigella, Listeria*, and *mycobacteria *as well as on *Saccharomyces cerevisiae*, *Candida albicans*, and spores of *Aspergillus niger* [21, 26]. In a study [43], the inactivation of *E. coli* and T4 and T7 phages after exposure to HILP was recorded. The evaluation of the effectiveness of HILP for the inactivation of *E. coli* and *L. innocua *in citric acid-disodium phosphate buffer solution was studied from Muñoz et al. [2]. However, the authors are unaware of any studies which directly compared the differences in susceptibility of *E. coli* and *L. innocua* to three different nonthermal light technologies (a NUV-vis light, a continuous UV, and a HILP light). So, the current study set out with two aims: (1) to test the relative susceptibility of two bacteria using three different light techniques; (2) to determine the effectiveness of each light equipment for inactivation of selected types of bacteria when different dosages are implemented.

**Figure 6:**
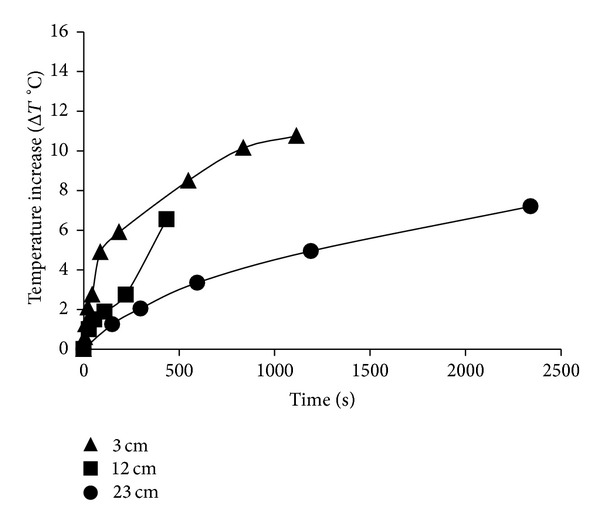
Mean Temperature increase (Δ*T*°C) for NUV-vis light technology at distances.

**Figure 7:**
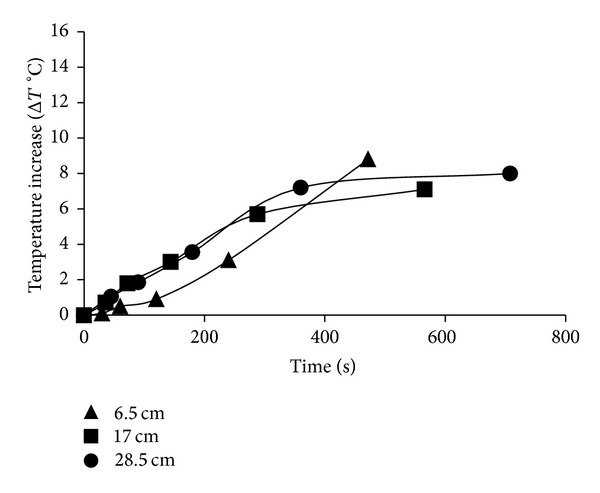
Mean Temperature increase (Δ*T*°C) for UV light technology at distances.

**Figure 8:**
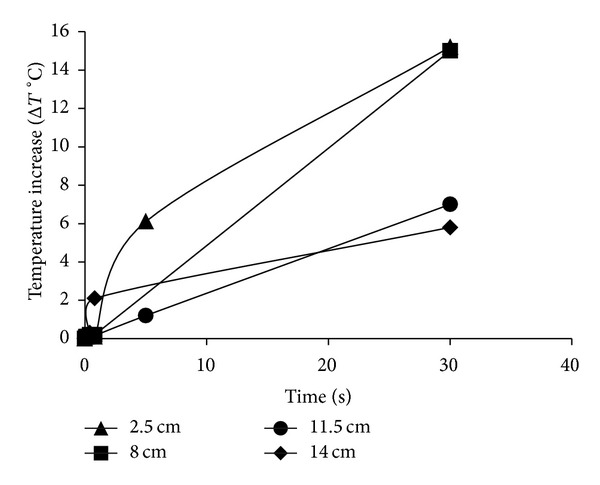
Mean Temperature increase (Δ*T*°C) for HILP light technology at distances.

Although longer treatment times resulted in significant temperature increases in all three light technologies, the dosages that were selected for this study did not result in temperature arise of more than 45°C (Figures 6, 7, and 8).

As the mechanism of inactivation by visible light is believed to be through the production of ROS, the susceptibility of both *E. coli* and *L. innocua* to ROS may play an important role in the inactivation of these organisms by NUV-vis light of 405 nm stimulates endogenous microbial porphyrin molecules to produce oxidizing reactive oxygen species (ROS), predominantly singlet oxygen (_1_O^2^) that damages cells leading to microbial death [20]. Specifically, 405 nm light has been shown to be capable of inactivating a range of predominantly nosocomial pathogens and also Gram-negative food-related pathogens [21, 23].

When NUV-vis light was implemented, *L. innocua* proved to be the most readily inactivated organism compared to *E. coli* (*P* < 0.05). Murdoch et al. [21] found that *L. monocytogenes* was most readily inactivated in suspension, whereas *S. enterica* was most resistant. They concluded that 395 ± 5 nm light inactivates diverse types of bacteria in liquids and on surfaces, in addition to the safety advantages of this visible (non-UV wavelength) light [21]. In addition, it is reported [26] that fungal organisms may be somewhat more resistant to 405 nm light than bacteria. In this study a correlation between dose (J/cm^2^) and microbes' inactivation was found. Other studies have reported that Gram-positive species, in general, were more susceptible to 405 nm light inactivation than Gram-negative species, which is generally consistent with the results obtained in the current study [44]. The prokaryotic bacteria also exhibit considerable variability in susceptibility with values, to achieve 5-log_10_ order reductions, as low as 18 J/cm^2^ with *Campylobacter jejuni* [45] but most typically around 50–300 J/cm^2^, with Gram-positive species being generally more susceptible than Gram-negatives [44]. Microbial inactivation by 405 nm light exposure has been found to be dose-dependent [21]. In applications where rapid inactivation is desirable, the use of a much higher power light source would significantly reduce the exposure times required for effective treatment. In our study, at the highest dosage (36 J/cm^2^), 1.37 log_10_ CFU/mL reduction was achieved for *E. coli *and a greater log reduction (2.74 log⁡_10_⁡ CFU/mL) was achieved for *L. innocua*. Our results are in accordance with another study [21], where they found that *L. monocytogenes* was completely inactivated at an average dosage of 128 J/cm^2^, whereas a 2.18 log⁡_10_⁡ reduction was achieved for *E. coli* at 192 J/cm^2^ dosage.

In the present study it was shown that, in order to achieve 2.66 log⁡_10_⁡ CFU/mL reductions for *E. coli* and 3.04 log⁡_10_⁡ CFU/mL for *L. innocua*, respectively, a dosage of 2.832 J/cm^2^ with continuous UV equipment was needed. However, the samples were not treated further due to the temperature arise. Our results are not in agreement with other studies [46] where better reductions (7.2 log⁡_10_⁡ CFU/mL reduction and 4.6 log⁡_10_⁡ CFU/mL reduction for *E. coli* and *L. innocua*, respectively, at 1.2 kJ/cm^2^) were achieved, perhaps due to different *E. coli* and *L. innocua* strains that were used. UV light creates mutated bases that compromise cell functionality, but bacteria have developed DNA repair mechanisms to restore DNA structure and functionality [47]. This phenomenon is reflected in the shape of the inactivation curves of our experiment [48].

The killing effects of HILP are caused by the rich and broad-spectrum UV content, the short duration, and the high peak power of the pulsed light produced by the multiplication of the flash power manifold [32, 49]. Other researchers found that a significant reduction of 3.6 log⁡_10_⁡ CFU/mL for *E. coli* *Κ*_12_ and 2.7 log⁡_10_⁡ CFU/mL for *L. innocua* (*P* < 0.001) was achieved with HILP (3.3 J/cm^2^) [2]. Our results are similar to that of study [2] as 2.57 log⁡_10_⁡ CFU/mL reduction for *E. coli* and 2.14 log⁡_10_⁡ CFU/mL reduction for *L. innocua* were achieved when 2.832 J/cm^2^ dosage was implemented. To the best of the authors' knowledge three studies referring to the application of high-intensity light pulses in a continuous system [42, 50, 51].

## 5. Conclusions

The results of the present study show that HILP treatments were more effective for the inactivation of both *E. coli* and *L. innocua. *Furthermore, this technology resulted in more rapid and extensive inactivation than either continuous UV-C and NUV-vis treatments. These observations associated with HILP may be attributable to the comparatively higher penetration depth and emission power compared to continuous UV-C and NUV-vis. Moreover it has a high peak power produced by the multiplication of the flash power manifold, producing a light intensity at least 100 times greater than that of other two light technologies during the same operating time. However, research must be performed in real food matrixes, as it is known that HILP light generates off flavors. It can be concluded that short treatment times for decontamination efficiency would be an important factor related to productivity in food industry. The findings presented here suggest the expansion of the aforementioned light technologies on food decontamination. Thus these alternative nonthermal disinfection light techniques could find potential applications for decontamination in the food industry.

## Figures and Tables

**Table 1:** Calculated exposure time (sec) of nonthermal light technologies at selected distances from the light source.

			Dose per treatment (J/cm^2^)										
			0.18	0.36	0.72	1.44	2.832	6	17.7	27	36	54	106.2
Distance from light source (cm)	3	NUV-VIS	6	11	22	45	88	186	548	836	1115	∗	∗
	12		28	55	110	221	435	∗	∗	∗	∗	∗	∗
	23		149	298	595	1190	2341	∗	∗	∗	∗	∗	∗
	6.5	UV	30	60	120	240	472	∗	∗	∗	∗	∗	∗
	17		36	72	144	288	566	∗	∗	∗	∗	∗	∗
	28.5		45	90	180	360	708	∗	∗	∗	∗	∗	∗
	2.5	HILP	NT	NT	NT	NT	0.8	NT	5	NT	NT	NT	30
	8		0.1	0.2	0.4	0.8	NT	NT	NT	NT	NT	30	NT
	11.5		NT	0.3	0.6	NT	NT	5	NT	NT	30	NT	NT
	14		0.2	0.4	0.8	NT	NT	NT	NT	30	NT	NT	NT

NUV-Vis: near UV-vis light; UV: ultraviolet light; HILP: high-intensity light pulses.

(∗) Samples that are not analyzed due to high temperature, NT: not tested samples.

Distance from light source (cm).

HILP was applied in pulses 360 *μ*s duration at a frequency of 3 Hz.

## Abbreviations

NUV-vis: Near ultraviolet/visible light
UV: Ultraviolet light
HILP: High-intensity light pulses
ROS: Reactive oxygen species
MRD: Maximum recovery diluent.
